# Development of an IoT Structural Monitoring System Applied to a Hypogeal Site

**DOI:** 10.3390/s20236769

**Published:** 2020-11-26

**Authors:** Alessio De Angelis, Francesco Santoni, Paolo Carbone, Manuela Cecconi, Alessia Vecchietti, Francesco Di Lorenzo

**Affiliations:** 1 Engineering Department, University of Perugia, 06125 Perugia, Italy; francesco.santoni@unipg.it (F.S.); paolo.carbone@unipg.it (P.C.); manuela.cecconi@unipg.it (M.C.); alessia.vecchietti@unipg.it (A.V.); 2 Polo Museale dell’Umbria, 06121 Perugia, Italy; francesco.dilorenzo-01@beniculturali.it

**Keywords:** accelerometers, structural monitoring, Internet-of-Things sensors, vibration sensing, sensors for cultural heritage monitoring

## Abstract

This paper describes the development of a distributed sensing system that can be disseminated in an environment of interest to monitor the vibration of a structure. This low-cost system consists of several sensor nodes and a central receiving node. All nodes are built using off-the-shelf electronic components. Each of the sensor nodes is battery-powered and equipped with a triaxial MEMS accelerometer, a wireless Long Range (LoRa) transceiver module for data transmission, a GPS module used for synchronization, and a microcontroller. The operation of the sensor node is validated by controlled laboratory tests where it is compared to a commercial reference accelerometer. Furthermore, the feasibility and potential benefits of the application of the proposed system to a structure in an archaeological site is investigated. Results show that the proposed sensor node could successfully monitor the vibration at several locations within the site. Therefore, it may be employed to detect the most relevant stresses to the structure, allowing for the identification of risks.

## 1. Introduction

Monitoring the actions and the induced stresses experienced by structures in historical and archaeological sites is crucial for risk assessment and cultural heritage conservation. Natural events, such as earthquakes, or anthropic actions, e.g., vehicular or railroad traffic and excavations, certainly lead to a change of the stress state in the structural system. The purpose of developing monitoring systems is to support maintenance, to identify the need for restoring actions, and to evaluate safety [1]. While monitoring is of fundamental importance for preventing damage and failure of critical infrastructures like bridges [2], it is also relevant for preserving cultural heritage assets.

In this context, considerable research activities have been devoted to structural health monitoring of monumental buildings. In [3], a vibration-based monitoring system was applied to a historical bell tower and the feasibility and benefits of distributed monitoring that is relatively low-cost are proven. In [4], an in-situ sonic testing campaign on adobe historical buildings is described and dynamic identification is performed, showing the need for restoration measures. Furthermore, static and dynamic monitoring of a bell tower in [5] allowed the gain of crucial knowledge for numerical modeling and preservation. A differential radar interferometry technique was used in [6] for assessing risks of both structural instability and land displacement. In [7], ambient vibration tests, i.e., tests where the vibration is caused by operating conditions and is not forced onto the structure, were performed on a heritage building. A survey of the application of ambient vibration tests and operational modal analysis to heritage structures is provided in [8], showing the considerable interest and relevance of such a topic. Finally, of great relevance are the hypogeal archeological sites, which are frequent in many Italian regions. In this case, due to the close interaction of the cavity with the surrounding soil, the preservation of the site from environmental and seismic risk also requires geotechnical aspects to be taken into due consideration. In the specific field of geotechnical engineering, it is well known that monitoring is extremely important for analyzing the behavior (and its evolution) of the geotechnical system to the variations of natural or anthropic external actions and/or boundary conditions. In addition, real-time automated measurement data, to be processed in a considerably short time, are essential to activate early warning systems, whether certain thresholds are exceeded. It follows that geotechnical monitoring results are essential for risk prevention and mitigation; a recent review of geotechnical monitoring and instrumentation is given in [9]. Two prototypal urban seismic monitoring systems based on the combination between Earthquake Observation (EO) and Structural Health Monitoring (SHM) systems have been recently installed in relevant public buildings in the historical city center of Catania and Acireale [10]. Such implementation can sensibly lower the potential impact of a destructive earthquake in an urban context. In [11], a long-term experimental study was conducted in an archaeological hypogeal site, the Mithraeum of the Baths of Caracalla in Rome, focusing on its microclimate and demonstrating the benefits of a hygrothermal monitoring system for risk assessment and preservation.

This paper describes research aimed at the development of a system for measuring vibration in a distributed fashion, intended for application to a cultural heritage hypogeal site. The area of interest is located at the outskirts of the city of Perugia (Central Italy), and belongs to a very important Etruscan archeological site, including several anthropic superficial cavities completely excavated in the subsoil, some of them very important from an archaeological point of view. It is known that the presence of natural or anthropic underground cavities (i.e., the geometry of the cavity, size and embedment depth) in the soil may affect the seismic ground response, see for example [12,13]. Hence, as a first goal, this research is motivated by the challenges in emerging remote and distributed monitoring scenarios. Such scenarios, in fact, require growing levels of energy efficiency to reduce the costs, impact, and risks associated with the usage of monitoring systems, to relax requirements for periodic maintenance and supervision [14], which is of major importance for cultural heritage monitoring applications. For this reason, the development of the proposed system is performed using Internet-of-Things (IoT) communication techniques, low-power microcontrollers, an event-driven strategy, and self-contained battery-powered electronics. The IoT framework, thanks to the convergence of sensing technologies, wireless communications, and internet connectivity, has the potential for being an integral part of many monitoring applications, and is currently being investigated for structural health monitoring [15]. At the same time, the novelty of the proposed system lies in the combination of the developed low-cost sensor platform with wireless communication capability and low-power operations, in conjunction with the specific application of vibration monitoring in cultural heritage sites with challenging access, such as the underground anthropic cavity we have considered in this study. In this paper, attention is mainly focused on the sensor designed, developed, and assembled throughout the present study. Experimental results prove that the developed low-cost sensing system is suitable for the considered monitoring application and provides results that are comparable to those of a much more expensive reference sensor.

## 2. Development of the Monitoring System

The proposed system is developed to enable distributed monitoring of acceleration in structures and geotechnical systems. Therefore, the system design requirements are low cost, fast prototyping, the connection of sensors to an online platform following the IoT paradigm, and flexibility for integrating different functions. Furthermore, the functional requirements of the sensor are the easy deployment in multiple positions for areal heterogeneity in the considered site, and the ability to detect relevant vibrations of a structure caused by anthropic activities, e.g., rail and vehicle traffic, and by earthquakes.

By applying a rapid prototyping approach, off-the-shelf components were selected in the design phase, thus limiting the development of analog electronic devices. This approach enabled the development of a working prototype in a short period of time. It also provided flexibility, since the modular architecture allows us to replace the specific sensor module with a different module for sensing another physical quantity, or to implement a multi-sensor platform.

**Figure 1:**
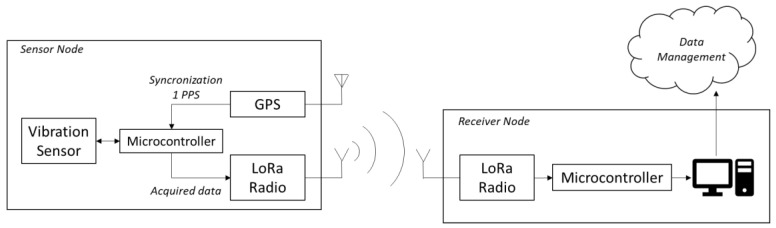
Block diagram depicting the architecture of the developed system.

As shown in Figure 1, the system is comprised of several self-contained and battery-powered sensor nodes and a receiver node. Each of the sensor nodes are equipped with a MEMS triaxial accelerometer, a low-power Long Range (LoRa) [16] radio module for data transmission, and a Global Positioning System (GPS) receiver, which is used for time synchronization through the 1-pulse-per-second (1PPS) signal. The choice of using the GPS receiver to implement the time synchronization functionality is motivated because it provides greater accuracy and coverage than other potential solutions, such as ultra-wideband radio [17] and inductive links [18], while promoting fast prototyping thanks to the wide availability of commercial modules.

In the developed system, each sensor node is synchronized independently using its own GPS receiver. This eliminates the need for exchanging synchronization messages between nodes. The synchronization of each sensor node is performed by reading the GPS navigation message sent from the satellites and received by the GPS receiver. This message contains a Coordinated Universal Time (UTC) timestamp. The internal real-time-clock (RTC) of the microcontroller in the sensor node is set to this UTC timestamp at the time instant denoted by the rising edge of the 1 PPS signal, which is generated by the GPS receiver.

To evaluate the performance achievable by this synchronization method, the time delay between the reception of the 1 PPS signal at two different sensor nodes was measured every second by an oscilloscope. The measurement was repeated 600 times, resulting in a maximum delay of 1.02 µs and a mean delay of 680 ns. These measured delays are suitable for the considered application, in which data are sampled every 10 ms, since they are considerably smaller than the sampling period. An on-board microcontroller is employed for managing and timing the operation of the node. The purpose of the receiver node is to gather data from all sensor nodes and transfer them to a data management system, making it available through an internet connection.

**Figure 2:**
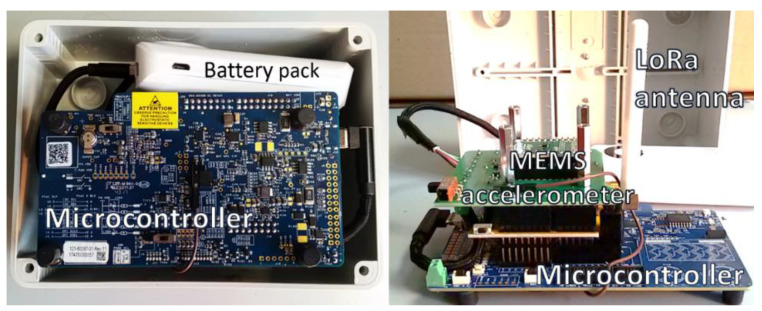
Pictures of the assembled electronic components and battery of the sensor node.

A picture of the system prototype is shown in Figure 2. The sensor and receiver nodes are implemented using a PSOC 6 microcontroller evaluation board by Cypress [19]. The acceleration sensor is a LIS2HH12 MEMS triaxial accelerometer by ST Microelectronics, with a resolution of 12 bits and a sampling frequency of 100 Hz [20]. This integrated accelerometer is configured with a range of ±2 g. In this configuration, the resolution of the employed accelerometer is approximately 0.001 g, thus fulfilling the functional requirements. The feasibility of using an accelerometer with the same sampling frequency, range, and resolution specifications is acknowledged in the literature in [21].

Furthermore, the wireless data transmission capability is provided by a Semtech SX1278 radio transceiver module, with a maximum transmitting power of +14 dBm and a receiver sensitivity of −148 dBm [22]. We conducted preliminary tests and experimentally verified that this module can establish a LoRa wireless communication with a range in the order of hundreds of meters or larger, even in non-line-of-sight conditions and in the presence of obstructions due to buildings. The sensor node is enclosed in an IP56 sealed electrical enclosure of size 150 × 110 × 70 mm.

The software development of the sensor node follows an event-driven approach. This reduces power consumption, improves energy efficiency, and thus promotes the easy dissemination of multiple nodes in the environment of interest. In particular, the node is normally in standby mode. In this mode, the microcontroller board and the LoRa module are off, whereas the accelerometer is always powered, and the microcontroller integrated chip is kept in a deep sleep mode.

When the acceleration measured by the accelerometer exceeds a predefined threshold, an interrupt signal is generated, which raises the level of a digital pin to a logic “high” level. When this pin goes high, it causes a “load switch” circuit [23] to connect the supply voltage to the rest of the system, which therefore transitions to the “on” state. Thanks to an internal First-In-First-Out (FIFO) memory, the accelerometer chip can keep track of the 32 latest samples acquired before the threshold-crossing event. After activation, the sensor node acquires acceleration data for a predefined time interval. Then, after this acquisition phase ends, acquired data are transmitted by the LoRa module and subsequently the node enters standby mode. To avoid packet collisions, every sensor node starts transmitting data after a specific delay, which is assigned univocally and programmed in the microcontroller software. Furthermore, to reduce the drift of the internal clock and thus preserve time-stamping accuracy, the sensor node is periodically activated by an alarm to allow GPS signal acquisition for synchronization.

In addition to the event-driven configuration described above, a continuous operation configuration is also implemented, which is selectable by the user. In the continuous operation configuration, the LoRa radio interface is disabled, and data are transmitted directly to a PC using a USB wired connection, which is also employed to power the sensor node. In this configuration, the PC runs a custom data acquisition software implemented in the MATLAB numerical computation environment, which performs the task of reading data via the USB port, storing it and processing it by numerical filtering, time-domain graphs, and a spectrogram. Moreover, the sensor can store data in the internal memory without connection to a PC. The microcontroller board has a 64 MB flash memory and therefore can store approximately 31 h of data at 100 Sa/s, since each sample requires 6 bytes. The continuous operation configuration is suitable for those applications where a real-time automated monitoring is required, e.g., for studying the effect of small vibrations due to traffic, railroads, or excavations.

To evaluate the energy requirements of the sensor node in the event-driven configuration, we measured its current consumption when powered by a 5 V rechargeable power-bank battery. In standby mode, the node requires a current of 2.6 mA, whereas during acquisition it consumes 144 mA of current. Furthermore, in the LoRa transmission phase, the current consumption is 290 mA, whereas in the GPS synchronization phase (cold start) it is 160 mA. By assuming one threshold-crossing event per day, a duration of the acquisition and LoRa transmission phases of 10 s and 20 s, respectively, and a GPS synchronization alarm every day with a cold start duration of 10 min, the average current consumption is about 3.77 mA in a 24-h time interval. Therefore, assuming a battery capacity of 10,000 mAh, battery duration is estimated to be about 110 days, which decreases to approximately 78 days in a worst-case scenario of 20 threshold-crossing events every day.

Finally, the data management functionality is implemented by a server application that manages a dynamic webpage and communicates with the receiver node to obtain the data. The application also handles data storage, retrieval, and graphical presentation.

## 3. Experimental Results

In this section, an experimental characterization of the proposed sensor node is presented. The goal of this characterization is to demonstrate the feasibility of the application of the proposed system to structural monitoring for the conservation of cultural heritage. A first characterization was performed in a controlled laboratory setting. Subsequently, as a case study, an additional characterization was carried out in an archeological site consisting of a hypogeal cavity and a masonry museum building.

### 3.1. Laboratory Tests

To validate the functionality of the proposed sensor node, we compare the data acquired by the node with those acquired by a reference instrument. This reference instrument is a commercial triaxial accelerometer, SA10 by SARA Electronic Instruments s.r.l. Such an accelerometer has a full-scale value of 1 g, sensibility 5 V/g, non-linearity <0.1%, cross-axis sensitivity <0.5%, and dynamic range greater than 165 dB. The signal from the reference accelerometer is digitized with a resolution of 24 bits and a sampling frequency of 100 Hz. The sampling frequency is the same as that of the proposed sensor node.

**Figure 3:**
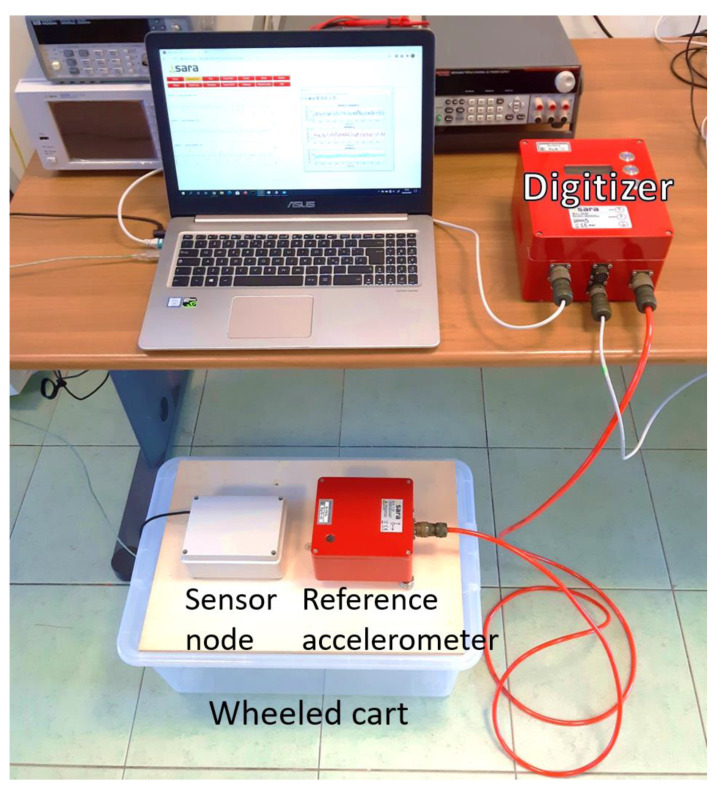
Laboratory test set up. The sensor node is placed on a wheeled cart (left side of the cart), alongside the reference accelerometer (right).

To perform the comparison, the sensor node and the reference accelerometer are placed on a wheeled cart as shown in Figure 3 so that they are subjected to the same acceleration, which is generated by applying an external force to the cart by hand. The y-axis of the sensor node and that of the reference accelerometer are both aligned along the direction of the fixed wheels of the cart, so that when applying an external force, the cart moves in the direction of the y-axis. The sensor node is setup in the continuous operation configuration, to be able to perform an uninterrupted acquisition.

An experiment was performed by initially applying a sequence of three short impulses to the cart. This sequence was repeated three times, with increasing force. Subsequently, a quasi-periodic oscillation was realized, by moving the cart back and forth. Again, this oscillation was repeated three times, with increasing force. 

A time alignment procedure was performed on the acquired records, to correct for the drift of the clock of the sensor node with respect to that of the reference accelerometer. The time-alignment procedure consists of first computing the cross-correlation of the two acquired records to estimate the time delay, followed by applying the dynamic time warping algorithm [24] to compensate for short-time clock fluctuations. Additionally, a second-order Butterworth low-pass digital filter with a cutoff frequency of 15 Hz was applied to both records, to reduce the impact of high-frequency noise. 

**Figure 4:**
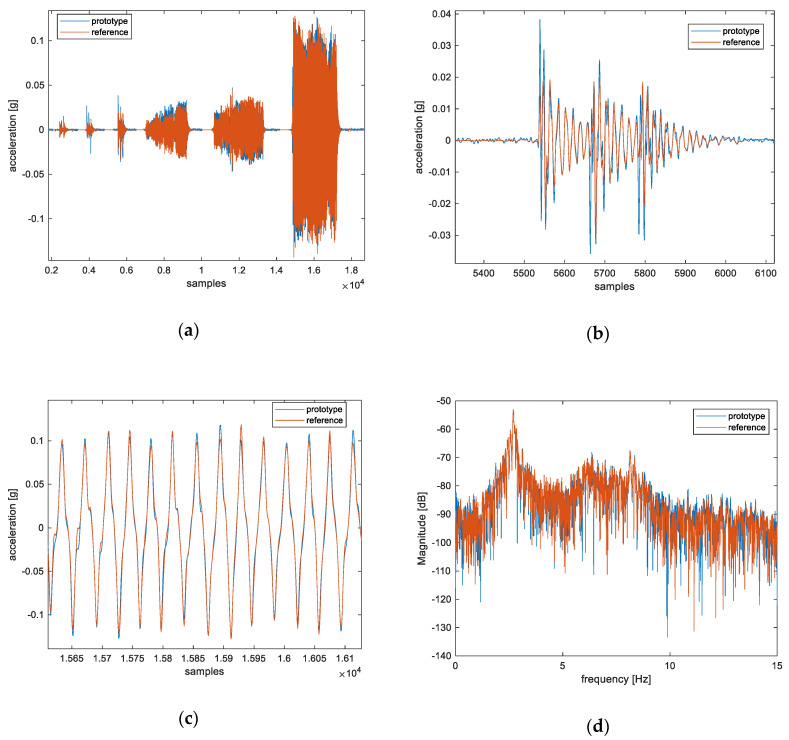
Experimental results from the laboratory test: (**a**) full record; (**b**) magnification of the section where the cart is subjected to three short impulses; (**c**) magnification of the section where a quasi-periodic acceleration input is provided to the cart; (**d**) DFT (discrete Fourier transform) of the full record with Hamming windowing.

The aligned data acquired during this experiment are shown in Figure 4a–c. From these figures, a good agreement can be noticed between the sensor node and the reference accelerometer, with a root-mean-square error of 0.0032 g, thus validating the behavior of the proposed sensor node. Furthermore, in Figure 4d, the discrete Fourier transform (DFT) is shown. From this figure, it is possible to notice that the sensor node and the reference accelerometer identify the same frequency components, with a main peak at approximately 2.7 Hz.

### 3.2. Field Tests

The feasibility of the proposed system for monitoring a cultural heritage structure was investigated by field tests. The goal of these field tests was exclusively to characterize the accelerometer used in the sensor node by comparing it to the reference. To achieve this goal, the tests were performed in the continuous operation mode, where the sensor node was powered via the USB connector by a PC, the GPS and LoRa module were disabled, and the battery of the sensor node was disconnected.

**Figure 5:**
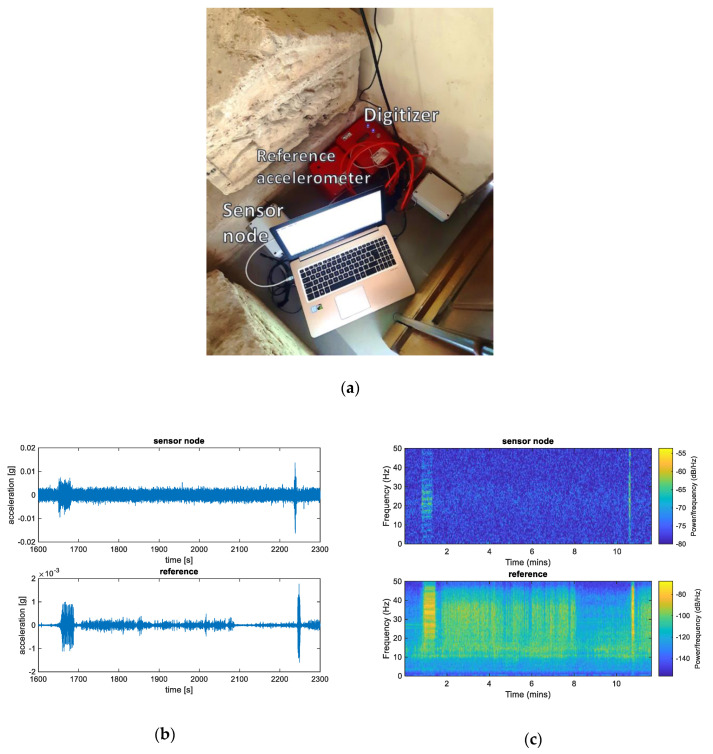
Experimental results from the field test: (**a**) photo of the set up in the museum building; (**b**) time domain plots of the x-axis acceleration; (**c**) spectrogram of the x-axis acceleration.

**Figure 6:**
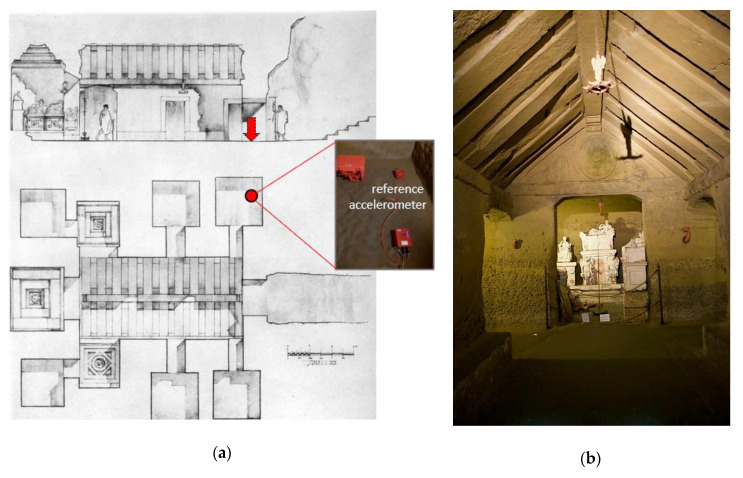
Volumni Hypogeum: cross section and plan view (**a**), adapted from [27]). Photo from the inside (**b**). The red symbol and the arrow in Figure 6a indicate the location of the reference accelerometer.

Initially, a preliminary measurement campaign was performed. The sensor node, in continuous operation configuration, was placed together with the reference accelerometer, as depicted in Figure 5a, on the floor of a museum masonry building at the archeological site of the Palazzone Etruscan necropolis, which dates back to the Hellenistic period (V sec B.C.). The Volumni Hypogeum (see Figure 5 and Figure 6) is the largest grave found in the necropolis, and belonged to the Velimna family. The archaeological studies started in 1840 and, from then, the necropolis has been studied from 1963 until today. A recent topographic survey and geological/stratigraphic identification of the archaeological site is documented in [25] while a preliminary assessment of the stability conditions of Volumni Hypogeum is described in [26]. The grave is entirely excavated into the ground; steep access stairs lead directly into a rectangular shape entrance hall (atrium) and to lateral rooms.

At the street level, near the building wall, approximately 10 m from the position where the sensor node was placed, a railroad track is present, with a level crossing and a road for vehicular traffic. During the measurement campaign, the passage of two trains was detected by the sensor node and reference accelerometer, as shown by the clearly visible signal peaks in Figure 5b at approximately the 1650 s and 2250 s marks. Furthermore, from the spectrogram of Figure 5c, it can be noticed that the train passage is characterized by a wideband frequency content.

**Figure 7:**
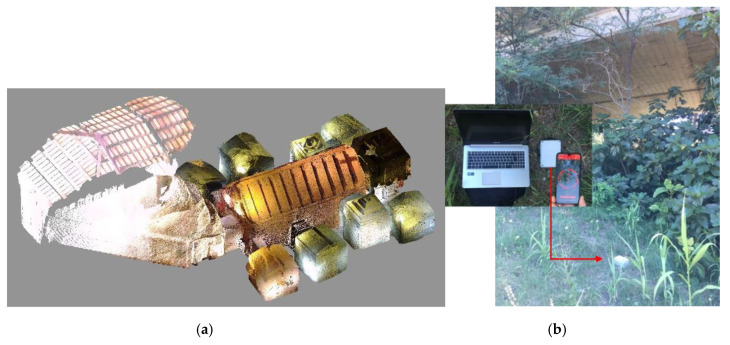

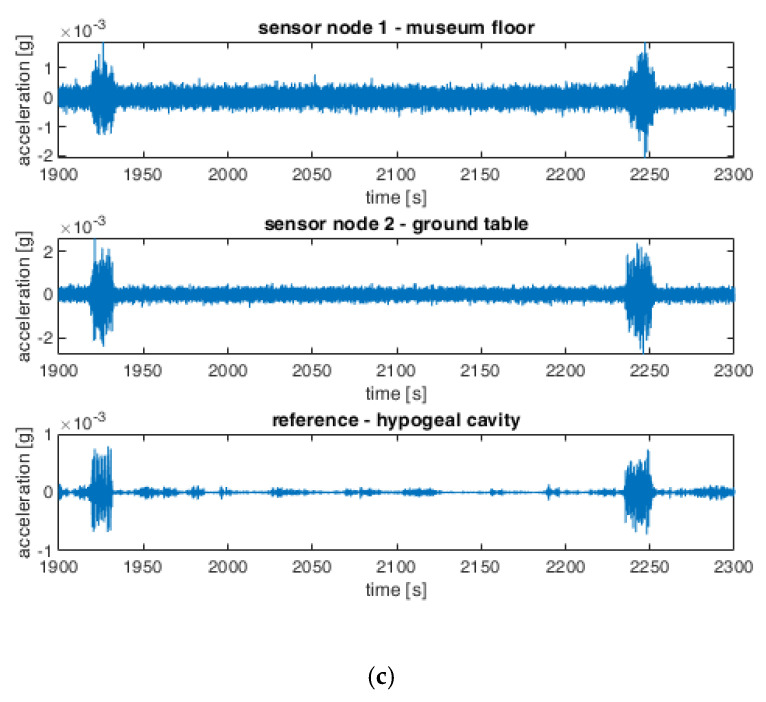
Measurement campaign at different positions in the “Volumni Hypogeum” archeological site. (**a**) Laser scan of the underground cavity, showing the structure of the cavity, stairs, and museum building [28]; (**b**) location of sensor node 2, at the ground table above the cavity; (**c**) data acquired by sensor node 1 on the museum floor, sensor node 2 at the ground table outside the museum above the cavity, and reference accelerometer, placed inside the cavity.

Subsequently, a second measurement campaign was performed on a different day at the same archeological site. The goal of this additional investigation was to characterize the vibration experienced inside the hypogeal cavity and compare it with that measured at street level to study the effect of the soil cover. In this additional campaign, the reference accelerometer was placed inside the hypogeal cavity, in a small lateral room (located on the right side when descending into the cavity though the access stairs, see Figure 6a). The cavity, whose cross section, plain view, and photo are shown in Figure 6, and whose structure is shown in Figure 7a, is excavated into sandy-gravel conglomerates deposits typical of the hill of Perugia [25].

For comparison purposes, a sensor node (denoted as sensor node 1) was placed inside the museum building in the same position as the preliminary measurement campaign, and another sensor node (denoted as sensor node 2) was placed outside the museum building at the ground table, directly above the chamber where the reference accelerometer was placed and in the proximity of a road overpass. 

To compare the results from the sensor nodes with the reference accelerometer, a calibration procedure was first performed. Such a procedure is necessary to compensate for the differences between the sensor node and reference accelerometer, which are mainly due to the construction methods, weight, and the mechanical effect of the sensor node’s enclosure. The calibration procedure is based on the data from the preliminary test, where sensors and reference were placed at the same position, and consists of the computation of a calibration factor by dividing the peak-to-peak amplitude of the sensor node measurements by that of the reference accelerometer. Then, the sensor node data are divided by this calibration factor, such that the peak-to-peak amplitude becomes the same as the reference accelerometer. Later, this calibration factor is applied also to the data from the second measurement campaign, so that the data are comparable.

Results from the second measurement campaign are shown in Figure 7c, where the data acquired by sensor nodes 1 and 2 are shown after applying the calibration factor. Two train passages are clearly detected by the three considered sensors at the 1920 and 2240 s marks, approximately. Furthermore, the amplitude of the acceleration measured by the reference accelerometer in the cavity is smaller than that of the two sensor nodes. It is also smaller than that measured during the preliminary campaign, when the reference accelerometer was placed on the museum floor, even if the type of trains was the same.

## 4. Discussion

The laboratory test results validate the proposed system by demonstrating that the sensor node provides data that is consistent with a reference instrument. The differences observed between the data acquired by the sensor node and those from the reference accelerometer, particularly evident in conditions of low acceleration amplitude, are due to the noise, the different accuracy level of the two accelerometers, the mechanical effect of the casing of the sensor node, and the imperfect alignment of the axes during the laboratory test. Nevertheless, these differences are still within acceptable bounds for the considered monitoring application, since the main components of the acceleration are correctly identified by the accelerometer in the developed sensor node, see e.g., Figure 4a.

Moreover, the preliminary field tests proved that the proposed system could monitor the major components of the acceleration experienced by structures, as shown by the results in Figure 5, and may detect potential risk situations. However, from Figure 5b, it is possible to notice that the MEMS accelerometer in the sensor node results in a worse signal-to-noise ratio than that of commercial-grade expensive accelerometers, such as the one used in this paper as a reference accelerometer. Additionally, the greater sensibility of the reference instrument results in more features in the acquired data, e.g., the time interval starting at approximately 2100 s in which the noise floor becomes very small, due to the closure of the level crossing and therefore the stop of vehicular traffic on the road. On the other hand, the presented results show that the proposed sensor node can monitor the most relevant accelerations, while keeping cost at a fraction of those of the reference accelerometers. Therefore, it could be easily deployed in the environment of interest in several positions, to obtain a more complete characterization of the vibrations and a higher degree of spatial diversity.

By comparing the laboratory test results of Figure 4a with the preliminary field test results of Figure 5b, it is possible to notice that the level of the signal in Figure 5b is lower (10-times difference) with respect to the reference system. This is due to the fact that the magnitude of the acceleration in the field tests (approximately 10^−3^ g) is much lower than that of the laboratory tests (approximately 10^−2^ g). In fact, we observed that the proposed system has a response that differs from that of the reference system when the acceleration level is low. This phenomenon is noticeable also in Figure 4a, in the case of the lower accelerations, but it is more evident in Figure 5b, where the acceleration is even lower. The cause of this phenomenon is due to construction and casing of the proposed MEMS accelerometer and associated electronics in the developed system, which has a much smaller mass than the reference accelerometer. The latter weighs 3 kg. However, this phenomenon is corrected by the calibration procedure described in Section 3.

Finally, from the results in Figure 7c, it is possible to observe that the acceleration measured inside the hypogeal cavity is attenuated with respect to that measured at street level. This suggests the effect of attenuation exerted from the soil mass at the top of the cavity, below the ground table (street level). Further research will concern the study of the geotechnical properties of the soil surrounding the cavity and its response to cyclic and dynamic actions, as well as the effect of the cavity on the seismic ground response.

Future work will be related to the extension of the functionality of the developed system, including the implementation of synchronization protocols using LoRa time-stamped packets, as described in the literature, see for example [29]. This would eliminate the need for GPS coverage at all sensor nodes, which might not be available in some applications. If some sensor nodes outside the in the network have access to GPS signal, then they can relay the timing information through LoRa time-stamped packets to those nodes that do not have GPS coverage.

## 5. Conclusions

A low-cost monitoring system was presented, which can measure the acceleration experienced by structures, to be implemented in earthquake monitoring networks or for structural health monitoring purposes. The system is comprised by multiple self-contained sensor nodes and a receiver node. The design of the sensor node is described, along with the design strategies used to meet the requirements of low power consumption and low cost. First, its operation is validated by means of laboratory tests by comparing it with a commercial reference accelerometer. The results confirm the correct operation of the developed node. Furthermore, the feasibility of the proposed system for monitoring a cultural heritage structure was investigated by field tests. These tests demonstrated that the developed low-cost system is able to monitor the vibration of the structure with spatial diversity, thus potentially detecting the most relevant stresses and promptly identifying the risks.
